# The Common *FTO* rs9939609 Polymorphism Interacts with Sleeping and Eating Windows to Affect Predisposition to Type 2 Diabetes

**DOI:** 10.3390/nu18030472

**Published:** 2026-02-01

**Authors:** Libi Kazarnovsky Nahshan, Danyel Chermon, Ruth Birk

**Affiliations:** Nutrition Department, Health Sciences Faculty, Ariel University, Ariel 40700, Israeldanyel@ariel.ac.il (D.C.)

**Keywords:** *FTO*, type 2 diabetes, eating window, fasting window, sleeping window, bedtime onset, sleeping quality, last meal timing, circadian rhythm

## Abstract

**Background:** The common fat mass and obesity-associated (*FTO*) gene variant rs9939609 has been linked to elevated risk of obesity and Type 2 diabetes mellitus (T2DM). Eating and sleeping windows gained clinical interest as factors in weight maintenance and have been linked to T2DM risk. **Objective:** To study the association and interaction between the common *FTO* rs9939609 variant and eating and sleeping windows to affect T2DM risk in a large community cohort. **Methods:** This cross-sectional study included 12,254 adult participants. Genetic, anthropometric, and lifestyle behaviors data including eating and fasting windows were analyzed. Logistic and linear regression models, as well as chi-square tests, were applied under additive, dominant, and recessive genetic models (adjusted for age, sex, and BMI). **Results:** Significant associations between *FTO* rs9939609 x eating and sleeping window interactions were demonstrated in relation to T2DM risk. Longer eating windows and later last meal timing were associated with an increased risk for T2DM under the additive model (OR = 1.029, 95% CI = 1.002–1.055, and OR = 1.066, 95% CI = 1.012–1.122, respectively), while longer fasting windows were found to be protective under additive model (OR = 0.972, 95% CI = 0.947–0.998). Later bedtime onset was associated with an increased risk for T2DM under additive model (OR = 1.101, 95% CI = 1.005–1.220). Hours of night sleep significantly interacted with *FTO* rs9939609 under additive genetic model. *FTO* rs9939609 risk allele carriers with prolonged sleeping windows (OR = 1.137, 95% CI = 1.039–1.354) and poorer sleeping quality (OR = 1.185, 95% CI = 1.038–1.354) had increased risk of T2DM. **Conclusions:** Eating and fasting windows, late last meal timing, hours of night sleep, late bedtime onset, and poorer sleep quality are significantly associated with T2DM risk among *FTO* rs9939609 risk carriers and may reflect metabolic vulnerability associated with *FTO* risk alleles. These findings highlight potential behavioral modification to attenuate genetic risk and provide evidence for actionable prevention strategies in genetically predisposed populations.

## 1. Introduction

Type 2 diabetes mellitus (T2DM) is a multifactorial metabolic disease caused by malfunction of the insulin action pathway [1]. T2DM is influenced by a complex interaction between genetic predisposition and environmental factors such as physical activity (PA) and diet [2,3]. The heritability of T2DM is considerable and estimated to range between 31 and 72% [4]. Although obesity remains one of the strongest risk factors for T2DM, up to 30% of the risk is attributable to other modifiable lifestyle factors, either directly or through their contribution to adiposity [5].

Genetic variants within the fat mass and obesity-associated gene (*FTO*) have been consistently linked to diabetes risk, independent of body mass index (BMI) [6,7,8]. A genome-wide association study (GWAS) confirms that rs9939609 variant located within the first intron of the *FTO* is associated with increased adiposity, insulin resistance, and impaired glucose homeostasis [9,10,11]. Although rs9939609 is an intronic variant, growing evidence demonstrates that this region functions as a regulatory element. Obesity-associated SNPs within intron 1 of *FTO* reside in enhancer-like regions that influence the expression of multiple genes involved in energy homeostasis and adipocyte biology [12,13,14]. Beyond transcriptional regulation, FTO functions as an N6-methyladenosine (m6A) RNA demethylase, thereby modulating post-transcriptional control of genes involved in adipogenesis, lipid metabolism, inflammation, and vascular dysfunction, all of which are relevant to the pathogenesis of obesity and T2DM [15,16].

Functional studies have shown that FTO expression in metabolic tissues such as skeletal muscle and pancreatic islets may influence insulin signaling, lipogenesis, and mitochondrial activity, suggesting a direct contribution to glucose dysregulation beyond its role in obesity [17,18,19]. Additionally, the *FTO* locus regulatory activity affects the expression of genes such as IRX3 and IRX5, which play key roles in adipocyte differentiation, thermogenesis, and metabolic regulation [12,13]. Accumulating evidence indicates that lifestyle behaviors, particularly those related to circadian alignment, can modify and interact with the metabolic effects of *FTO* variants [20,21,22,23,24,25,26]. FTO has been shown to modulate circadian biology by inhibiting CLOCK–BMAL1 induced transcription and interacting with cryptochromes, thereby linking FTO activity to the core molecular clock machinery [27]. Furthermore, evidence accumulated over the last decade indicates that the pace of the circadian clock is controlled by m6A-dependent RNA processing. For instance, suppression of m6A methylation and its impact on RNA processing are sufficient to slow down the circadian clock [28,29]. Notably, FTO is highly expressed in the hypothalamus, where it regulates energy intake and feeding behavior. Carriers of the rs9939609 A allele exhibit dysregulated ghrelin signaling and impaired postprandial appetite suppression. Furthermore, the most prevalent SNP rs9939609 risk allele was found to be associated with increased *FTO* mRNA levels in adipose tissue and peripheral blood mononuclear cells and skin biopsies [30,31,32].

Emerging lifestyle factors related to circadian alignment have gained increasing attention as potential modulators of glucose metabolism and cardiometabolic health [1,3,26,33]. Given the involvement of FTO in circadian regulation and appetite control, behavioral timing may represent interface through which genetic susceptibility translates into metabolic risk. Disrupted sleep or irregular eating patterns have been shown to impair insulin sensitivity, increase glucose levels, and disturb hormonal rhythms regulating metabolism [5,34,35]. Despite these advances, limited evidence exists regarding direct gene–environment factors such as eating and sleeping windows interactions in general and between *FTO* rs9939609 and behavioral factors in relation to T2DM in particular [2,3]. Previous studies have primarily examined the main effects of FTO or lifestyle factors separately, with few exploring their combined influence on metabolic outcomes [17,20,21,22].

The timing and regularity of sleep and eating behaviors are increasingly recognized as critical determinants of glycemic control and T2DM risk. The American Heart Association highlights that greater rhythmicity in lifestyle behaviors, including consistent sleep and meal timing, is associated with lower risk of glycemic dysregulation and T2DM, while irregular patterns are linked to increased risk, independent of traditional risk factors [36]. The American Diabetes Association emphasizes that both short (<6 h) and long (>9 h) sleep durations, poor sleep quality, and evening chronotype confer a significantly higher risk of T2DM [37]. Recent evidence suggests that the adverse metabolic effects of the *FTO* risk allele may be amplified by excessive fuel availability [38]. Circadian misalignment occurs when the body’s internal clock is out of sync with external environmental cues (like light–dark cycles, sleeping or eating patterns), which leads to hormonal imbalances, impaired glucose metabolism, and gut microbiome dysbiosis [39], resulting in excessive energy storage and increased risk of metabolic disorders such as obesity and T2DM.

We aimed to study the association between *FTO* rs9939609, modifiable lifestyle behaviors such as eating and sleeping behaviors, and T2DM risk in a large Israeli adult cohort. We hypothesized that specific lifestyle behaviors, particularly those related to circadian and behavioral timing, may modify the genetic susceptibility to T2DM.

## 2. Materials and Methods

### 2.1. Participants

The cross-sectional study included data (Israeli registry database (#700068969) of Lev Hai Genetics Ltd., (Tel-Aviv, Israel) 21 December 2021–May 2023) of 12,254 Israeli adults (64% female) with a mean age 56.4 ± 15.7 years. Non-identifiable genetic data were used. The study was approved by the ethical committee of Ariel University (#AU-HEA-RB-20220214). Participants under the age of 18 years, having a genetic disease or missing genetic, lifestyle, and anthropometric data were excluded from the study.

### 2.2. SNP Selection and Hardy–Weinberg Equilibrium (HWE)

The single nucleotide polymorphism (SNP) *FTO* rs9939609 was selected for this analysis based on its well-established association with obesity, insulin resistance, and T2DM reported in multiple GWAS across various populations [9,10]. The selection was further supported by previous meta-analyses demonstrating a consistent effect of the A risk allele on metabolic traits and glucose regulation [8,9,26,33]. Genotype frequencies were tested for Hardy–Weinberg equilibrium (HWE) using a chi-square (χ^2^) test with 1 degree of freedom. The observed genotype distribution did not deviate significantly from HWE (*p* > 0.05), confirming the reliability of the genotyping process.

### 2.3. Lifestyle Variables

Participants filled in an online questionnaire regarding anthropometric measurements (height and weight), and their lifestyle habits including eating and fasting windows (hours between first and last meal, and fasting window, time of last meal), and sleep behaviors (bedtime onset, sleep duration).

Sleep duration and bedtime onset were analyzed as continuous and categorical variables. Categorical brackets were defined by tree analysis combined with scientific recommendations emphasizing the metabolic and circadian advantages of adequate sleeping duration (7–8 h) and earlier bedtime [40,41]. Bedtime onset categorized into before 21:00, 21:00–23:00, and after 23:00.

Eating and fasting windows were derived from participants’ self-reported meal timing data. Respondents indicated whether they consumed food during six predefined eating occasions (breakfast, morning snack, lunch, afternoon snack, dinner, and late-night meal) and, for each reported meal, selected an approximate time range (e.g., breakfast: before 6:00, 6:00–8:00, 8:00–10:00, etc.). The eating window was defined as the time interval between the first and last reported meal, and the fasting window was calculated as the remaining hours in a 24 h period. Both eating window and fasting window durations were examined as continuous and categorical variables. Categorical brackets were constructed using decision-tree-derived cut-offs and supported by prior literature to reflect meaningful behavioral ranges to facilitate interpretation and to reflect meaningful behavioral ranges [42].

### 2.4. Statistical Analysis

Categorical variables were compared between participants with and without T2DM using chi-square (χ^2^) tests, presented as n (%). Continuous variables were analyzed using *t*-tests, presented as mean ± standard deviation (SD).

Binary logistic regression models were applied to assess the associations between *FTO* rs9939609 genotypes and T2DM risk under additive, dominant, and recessive models. OR was calculated using the TT genotype as the reference group. In the dominant model, carriers of at least one A allele (AT + AA) were compared with TT, whereas in the recessive model, AA homozygotes were compared with TT + AT. Each model was adjusted for potential confounders including age, sex, and BMI.

To examine gene–environment interactions, logistic regression analyses were conducted including interaction terms (*FTO* rs9939609 × lifestyle variable). Interaction models evaluated whether the association between genotype and T2DM risk was modified by lifestyle parameters such as eating/fasting window, sleeping parameters, etc. Variables were analyzed in continuous form as well as categorical form using literature-based knowledge or SPSS tree-derived category structures when applicable. Sensitivity analyses were conducted using regression models with and without BMI adjustment. The consistency of main genetic effects and gene–lifestyle interaction estimates across adjustment strategies was examined to evaluate the influence of BMI on the observed associations.

Only interaction terms included in the final regression models are presented in the results. Interactions were analyzed by the additive models, with further analysis using other models when additive model was not significant. The significance of each interaction was determined based on the *p*-value of the interaction term in the logistic regression model and was set at *p* < 0.05. Statistical analyses were performed using SPSS 29.0 for Windows (SPSS Inc., Chicago, IL, USA).

## 3. Results

### 3.1. Participant Characteristics

This cross-sectional study included 16,274 Israeli adult participants (64% female), with a mean age of 56.4 ± 15.7 years. Population characteristics are presented in Table 1. Participants with T2DM (*n* = 933) were significantly older and had higher BMI compared to those without T2DM (*p* < 0.001 for both). Females were less represented in the T2DM group (56%) compared to the non-T2DM group (70%, *p* < 0.001).

Lifestyle-related differences were also observed between groups. Participants with T2DM reported longer noon sleep duration (*p* < 0.01), lower sleep quality (*p* < 0.01), and later eating windows (*p* < 0.001), while fasting windows were significantly shorter (*p* < 0.001).

### 3.2. FTO rs9939609 Genotype Frequency and Association with T2DM

#### 3.2.1. FTO rs9939609 Genotype Frequency and T2DM

Among participants, 27.3% carried the TT genotype, 48.7% were heterozygous (AT), and 24.0% were homozygous for the risk allele (AA). Regression models adjusted for age, sex, and BMI indicated that carriers of the risk allele had a significant increased risk of T2DM under all genetic models: additive (OR= 1.16; 95% CI: 1.057–1.28, *p* = 0.002), recessive (OR =1.23; 95% CI: 1.056–1.44, *p* = 0.008), and dominant (OR = 1.218; 95% CI: 1.036–1.43, *p* = 0.017) (Table 2). These findings suggest a dose-dependent effect, consistent with a recessive model of inheritance.

#### 3.2.2. Association of Lifestyle Variables with T2DM

Several lifestyle variables were significantly associated with T2DM status in the adjusted logistic regression models (Table 3). Older age and higher BMI were strongly associated with increased odds of T2DM (OR = 1.07 per year, 95% CI: 1.06–1.08; OR = 1.03 per kg/m^2^, 95% CI: 1.02–1.04; both *p* < 0.001). Patterns of food timing also showed significant associations. Eating at night was linked with higher T2DM risk (OR = 1.13, 95% CI: 1.05–1.21; *p* = 0.001), and a greater number of meals per day was similarly associated with elevated risk (OR = 1.13, 95% CI: 1.07–1.18; *p* < 0.001). Earlier first-meal timing was protective (OR = 0.94, 95% CI: 0.91–0.97; *p* < 0.001), while later last meal timing increased T2DM risk (OR = 1.03, 95% CI: 1.01–1.06; *p* = 0.004). Longer eating window duration was associated with higher T2DM odds (OR = 1.04 per hour, 95% CI: 1.03–1.06; *p* < 0.001), whereas longer fasting windows were protective (OR = 0.96 per hour, 95% CI: 0.94–0.98; *p* < 0.001). Correspondingly, categorical analyses showed that wider eating windows significantly increased T2DM odds (OR = 1.44, 95% CI: 1.27–1.63; *p* < 0.001), while longer fasting window categories were strongly protective (OR = 0.70, 95% CI: 0.61–0.79; *p* < 0.001). Sleep-related behaviors were also associated with T2DM risk. Higher sleep-quality scores were linked to reduced odds of T2DM (OR = 0.84, 95% CI: 0.76–0.93; *p* < 0.001).

### 3.3. Gene–Environment Interactions

#### 3.3.1. Eating and Fasting Window Interact with *FTO* rs9939609 to Predispose to T2DM

Eating and fasting window categories were defined using cut-off points derived from decision tree analysis, which were subsequently evaluated in the context of previously published evidence. Eating window length significantly interacted with *FTO* rs9939609 under additive genetic model (*p* = 0.033). Carriers of the risk A allele had an increased risk of T2DM with prolonged eating windows as a continuous measure. Eating windows, expressed in tertiles (ranging between <8, 8–14, and >14 h), were associated with higher T2DM risk among A risk allele carriers under dominant model. Fasting window duration showed significant interaction with *FTO* rs9939609 under additive model (*p* = 0.033). Carriers of the risk A allele were at higher risk for T2DM when fasting windows were expressed in tertiles (ranging between <9, 9–15, and >15 h), whereas TT individuals were less affected. Timing of the last meal of the day significantly interacted with *FTO* rs9939609 under the dominant model (*p* = 0.015) (Table 4).

#### 3.3.2. Sleeping Window and Quality Interaction with *FTO* rs9939609 on T2DM Risk

Sleep quality significantly interacted with *FTO* rs9939609 under additive models (*p* = 0.012). Bedtime onset significantly interacted with *FTO* rs9939609 in additive model (*p* = 0.039). When categorized, later bedtimes conferred elevated T2DM risk particularly among risk allele carriers. Night sleep duration (hours) significantly interacted with *FTO* rs9939609 under additive model (*p* = 0.005; Table 4).

## 4. Discussion

This study examined the relationship between *FTO* rs9939609, lifestyle factors, and T2DM risk in a large Israeli cohort of 12,254 adults. Carriers of the *FTO* A risk allele exhibited higher odds of T2DM, supporting the established role of *FTO* in metabolic regulation [8], across diverse populations [8,43], including the Israeli and Middle Eastern cohorts [44,45]. The elevated risk for T2DM in *FTO* risk carriers is partly mediated by the increased adiposity. However, meta-analyses and large cohort studies indicate an independent effect of *FTO* on T2DM susceptibility [8,43,46], likely through direct effects on glucose metabolism and insulin sensitivity [43]. For instance, FTO expression in human islet cells is not associated with BMI, and increased FTO levels in muscle have been linked to altered insulin signaling, enhanced lipogenesis, oxidative stress, and mitochondrial dysfunction during T2DM [18].

Previous studies have shown that time-restricted eating window (e.g., 8–10 h) and longer fasting window can improve insulin sensitivity and glycemic control in adults with T2DM [3,8,47]. However, to date, for the best of our knowledge, genetic predisposition in this context was not studied. Our novel findings demonstrate significant association between prolonged eating windows and shorter fasting windows with higher T2DM risk among *FTO* rs9939609 A allele carriers.

Adjustment for BMI attenuated the association between *FTO* rs9939609 and T2DM, consistent with adiposity acting as a partial mediator of the genetic effect. In contrast, the gene–lifestyle interactions of *FTO* rs9939609 with eating window and sleep related variables remained largely unchanged after BMI adjustment. This pattern suggests that while adiposity contributes to overall T2DM risk, the observed gene–behavior interactions are not solely explained by BMI and may reflect BMI-independent pathways related to behavioral timing or circadian regulation. Beyond the interaction detected in the regression models, exploratory decision tree analyses suggested that the predisposition of *FTO* rs9939609 may be concentrated within specific behavioral ranges. For eating window duration, the tree identified the following categories: ≤8 h, 8–14 h, and >14 h. Those intervals correspond to the median daily eating window in United States adults of 14 h [48], and to the eating window of 8 h which is linked to metabolic benefits including insulin resistance improvements [46]. Importantly, the gene–environment interaction emerged specifically within the mid-range of 8–14 h, where *FTO* A allele carriers showed higher T2DM prevalence compared to TT individuals. In contrast, at eating windows >14 h and ≤8 h, T2DM prevalence was high and low, respectively, for overall population. These findings imply that *FTO*-related susceptibility is amplified when eating window duration corresponds to specific time window (8–14 h); above this time window the risk for T2DM is high for the overall population (regardless of *FTO* susceptibility).

In relation to T2DM, under circadian misaligned conditions, such as those induced by irregular sleep and eating patterns, lipid metabolism in skeletal muscle may be disrupted, including phosphatidylcholine species [49,50]. Carriers of the FTO rs9939609 risk allele exhibit genotype-dependent changes in serum phosphatidylcholine metabolites, which are established metabolic markers of obesity and T2DM [51]. It is therefore plausible that the coexistence of FTO risk allele carriage and circadian disruption contributes to alterations in skeletal muscle lipid composition, potentially reducing insulin sensitivity and impairing glucose homeostasis.

Timing of the last meal appeared to modulate T2DM risk through its interaction with *FTO* rs9939609, supporting evidence that late-night eating can impair metabolic regulation through circadian disruption. Aligned with our results, the American Heart Association states that eating the last meal after 9:00 pm, compared to before 8:00 pm, is associated with a 28% higher risk of T2DM [36]. Nonetheless, recent interventional studies and randomized controlled trials have not demonstrated that modifying meal timing in individuals who are carriers of the *FTO* risk allele leads to measurable improvements in glycemic control or T2DM incidence [26,52]. Both *FTO* risk allele carriers and non-carriers experienced similar improvements in fasting blood glucose, insulin levels, and insulin resistance after dietary interventions, with no statistically significant gene–diet interaction for glycemic outcomes [52].

Sleep duration and bedtime onset emerged in a significant association with *FTO* rs9939609 and T2DM risk. Longer night sleep and later bedtime onset were associated with increased T2DM risk among A risk allele carriers. While insufficient sleep is a well-established metabolic risk factor, the current findings suggest that within the recommendation [37], prolonged sleeping hours [27,30] may reflect metabolic vulnerability associated with *FTO* risk alleles, while above this time window the risk for T2DM is high for the overall population. Tree analysis clarified that the genotype effect appeared specifically within 6–8 h, where *FTO* A allele carriers had higher T2DM prevalence. In contrast, very short sleep (<5 h) and prolonged sleep (≥9 h) displayed elevated diabetes prevalence overall, but without genotype-based splits suggesting that at exposure extremes the behavioral factor predominates the *FTO* genetic predisposition. Our findings align in part with Li et al. (2021), who observed that both short and long sleep durations, as well as late chronotype, were associated with increased risk of T2DM in a large prospective UK cohort [34]. They showed that these associations persisted across different levels of polygenetic risk, suggesting that sleep and circadian behaviors independently contribute to diabetes susceptibility [34,53,54].

Subjective poorer sleep quality was associated with higher T2DM risk, and genetic predisposition interaction was observed in the logistic regression model. In the decision tree analyses, poorer sleeping quality significantly elevated T2DM risk regardless of *FTO* genetic background, while fair or good sleep quality interacted with *FTO* predisposition, where carriers of *FTO* risk allele exhibited nearly double the T2DM prevalence. This pattern, together with the observation of increased risk within the recommended range of sleep duration, may be consistent with reports linking *FTO* risk alleles to obstructive sleep apnea [55]. Furthermore, subjective sleep quality ratings do not necessarily reflect the frequency of short awakenings or objective sleep depth [56], and thus individuals with different genotypes may experience or interpret “good” or “fair” sleep differently.

Our findings should be interpreted cautiously as the cross-sectional nature of the study precludes causal inference. Reverse causality is plausible, particularly given that metabolic dysregulation and T2DM diagnosis may lead to behavioral changes in eating patterns and sleep habits; it is also plausible that carriers of the *FTO* rs9939609 risk allele are inherently more prone to circadian dysregulation. Evidence suggests that *FTO* is involved in central clock regulation, appetite signaling, and behavioral timing [27,32], which may predispose genetically susceptible individuals to irregular eating and sleep patterns. Moreover, although infancy-related exposures were not examined in the present study, the postnatal period represents a vulnerable window for circadian clock programming, during which feeding patterns act as chronobiological signals [57]. Disruptions during this early period, such as formula feeding compared with breastfeeding, may contribute to long-term circadian and metabolic vulnerability [58]. Barragán et al. emphasized the clinical relevance of meal and sleep timing in the context of genetically driven obesity risk in Mediterranean population [59]. Specifically, Barragán et al. found significant gene–sleep interactions between the midpoint of sleep and *FTO*-rs9939609 polymorphism which played a role in determining obesity phenotypes [59]. Our results extend these observations to T2DM, indicating that circadian-related eating and sleeping patterns may represent clinically relevant factors for personalized metabolic risk stratification among *FTO* rs9939609 risk allele carriers. Although several gene–lifestyle interactions reached statistical significance, the observed effect sizes were modest, which is expected given the multifactorial nature of circadian regulation and behavioral timing. Such effects are therefore likely to be most relevant at the population level and over longer time horizons, rather than as deterministic predictors at the individual level. From a clinical perspective, these findings support assessing behavioral timing and circadian-related lifestyle patterns as part of metabolic risk stratification, particularly among individuals with known genetic susceptibility.

The study’s strengths include its large sample size, detailed assessment of sleeping and eating behaviors, and the examination of multiple genetic models. However, several limitations should be noted: lifestyle behaviors were self-reported and therefore subject to recall and reporting bias; the cross-sectional design precludes causal inference; and most lifestyle variables were self-reported and therefore subject to recall and perception bias.

## 5. Conclusions

The common *FTO* rs9939609 variant is significantly associated with elevated T2DM risk. Longer eating window and shorter fasting window, the timing of the last meal, typical bedtime onset, and habitual sleep duration and quality may all play a role in shaping how genetic predisposition affects T2DM. Certain behavioral choices, such as eating windows within approximately 8–14 h, early last-meal timing, and bedtime earlier than 23:00, may represent intervals in which genotype related differences become more pronounced. Our results underscore potential avenues for future research exploring how circadian-behavior-linked factors may interact with genetic risk to influence metabolic health.

## Figures and Tables

**Table 1:** Baseline characteristics of the study population by T2DM status.

Variable *	Total (*n* = 12,254)	No T2DM (*n* = 11,321)	T2DM (*n* = 933)	*p*-Value
Age (years)	56.4 ± 15.7	55.8 ± 15.3	67.0 ± 9.8	<0.001
Sex (females, *n* [%])	10,417 (64%)	7925 (70%)	523 (56%)	<0.001
BMI (kg/m^2^)	31.5 ± 6.1	31.2 ± 6.0	32.7 ± 6.0	<0.001
Night sleeping (Hours)	2.36 ± 0.99	2.37 ± 0.99	2.30 ± 1.04	0.004
Noon sleeping (Hours)	1.43 ± 0.72	1.40 ± 0.70	1.66 ± 0.83	<0.01
Sleeping quality (score 1–3)	2.25 ± 0.84	2.26 ± 0.84	2.10 ± 0.82	<0.01
Eating window (Hours)	10.0 ± 4.1	9.95 ± 4.13	10.82 ± 3.95	<0.001
Fasting window (Hours)	14.0 ± 4.1	14.05 ± 4.13	13.18 ± 3.95	<0.001

* Values are presented as mean ± SD for continuous variables and *n* (%) for categorical variables. *p*-values derived from *t*-tests or χ^2^ tests as appropriate.

**Table 2:** FTO rs9939609 genotype frequencies and association with T2DM *.

Genotype	Overall Population *n* = 12,254	T2DM *n* = 933	Non-T2DM *n*= 11321	Dominant Model	Recessive Model	Additive Model
TT	3350 (27.3%)	218 (23.4%)	3132 (27.7%)	OR = 1.218 (1.036–1.43) *p* = 0.017	OR = 1.234 (1.056–1.44) *p* = 0.008	OR = 1.164 (1.057–1.28) *p* = 0.002
AT	5969 (48.7%)	455 (48.8%)	2675 (23.6%)			
AA	2935 (24%)	260 (27.9%)	5514 (48.7%)			

* ORs adjusted for age, sex, and BMI, were calculated using TT genotype as the reference group. In the dominant model, carriers of at least one A allele (AT + AA) were compared with TT. In the recessive model, AA homozygotes were compared with TT + AT. The additive model assumed a per-A allele effect.

**Table 3:** Associations of lifestyle variables with T2DM *.

Variable	OR (95% CI)	*p*-Value
Age (per year)	1.07 (1.06–1.08)	<0.001
BMI (per kg/m^2^)	1.03 (1.02–1.04)	<0.001
Eating at night (score 1–4)	1.13 (1.05–1.21)	0.001
Number of meals/day	1.13 (1.07–1.18)	<0.001
First meal timing (hours)	0.94 (0.91–0.97)	<0.001
Last meal timing (hours)	1.03 (1.01–1.06)	0.004
Eating window (hours)	1.04 (1.03–1.06)	<0.001
Fasting window (hours)	0.96 (0.94–0.98)	<0.001
Eating window categories	1.44 (1.27–1.63)	<0.001
Fasting window categories	0.70 (0.61–0.79)	<0.001
Sleep quality (score 1–3)	0.84 (0.76–0.93)	<0.001

* OR and 95% CI were derived from multivariable logistic regression models adjusted for age, sex, and BMI, unless otherwise specified. Continuous variables were modeled per unit increase.

**Table 4:** Interactions between FTO rs9939609 and lifestyle behaviors on T2DM risk *.

Variable	Genetic Model	β	OR (95% CI)	*p*-Value
Bed time onset	Add	0.102	1.101 (1.005–1.220)	0.039
Bed time onset categories	Add	0.192	1.212 (1.030–1.426)	0.021
Hours of night sleeping	Add	0.129	1.137 (1.039–1.245)	0.005
Sleeping quality	Add	0.170	1.185 (1.038–1.354)	0.012
Eating window	Add	0.028	1.029 (1.002–1.055)	0.033
Fasting window	Add	−0.028	0.972 (0.947–0.998)	0.033
Eating window categories	Dom	0.318	1.375 (1.028–1.838)	0.032
Fasting window categories	Dom	−0.318	0.727 (0.544–0.973)	0.032
Last meal timing	Dom	0.064	1.066 (1.012–1.122)	0.015

* β coefficients, OR, and *p*-values represent the interaction terms between *FTO* rs9939609 and each lifestyle variable, derived from multivariable logistic regression models adjusted for age, sex, and BMI. The additive model assumes A allele effect, while the dominant model compares carriers of at least one A allele (AT + AA) with TT.

## Data Availability

No new data were created or analyzed in this study.

## Abbreviations

The following abbreviations are used in this manuscript: Type 2 diabetes mellitus (T2DM); Physical activity (PA); Fat mass and obesity-associated gene (FTO); Body mass index (BMI); Single nucleotide polymorphism (SNP); Genome-wide association studies (GWAS); Hardy–Weinberg equilibrium (HWE); Standard deviation (SD).
